# A Multiscale Spatio-Temporal Convolutional Deep Belief Network for Sensor Fault Detection of Wind Turbine

**DOI:** 10.3390/s20123580

**Published:** 2020-06-24

**Authors:** Hong Wang, Hongbin Wang, Guoqian Jiang, Yueling Wang, Shuang Ren

**Affiliations:** 1 School of Electrical Engineering, Yanshan University, Qinhuangdao 066004, China; hongw329@163.com (H.W.); jiangguoqian@ysu.edu.cn (G.J.); yuelingw@ysu.edu.cn (Y.W.); 2 China Academy of Information and Communication Institute, Beijing 100089, China; renshuang@caict.ac.cn

**Keywords:** fault detection, classification, wind turbine sensor, multivariate time series, multiscale spatio-temporal convolutional deep belief network, deep learning

## Abstract

Sensor fault detection of wind turbines plays an important role in improving the reliability and stable operation of turbines. The supervisory control and data acquisition (SCADA) system of a wind turbine provides promising insights into sensor fault detection due to the accessibility of the data and the abundance of sensor information. However, SCADA data are essentially multivariate time series with inherent spatio-temporal correlation characteristics, which has not been well considered in the existing wind turbine fault detection research. This paper proposes a novel classification-based fault detection method for wind turbine sensors. To better capture the spatio-temporal characteristics hidden in SCADA data, a multiscale spatio-temporal convolutional deep belief network (MSTCDBN) was developed to perform feature learning and classification to fulfill the sensor fault detection. A major superiority of the proposed method is that it can not only learn the spatial correlation information between several different variables but also capture the temporal characteristics of each variable. Furthermore, this method with multiscale learning capability can excavate interactive characteristics between variables at different scales of filters. A generic wind turbine benchmark model was used to evaluate the proposed approach. The comparative results demonstrate that the proposed method can significantly enhance the fault detection performance.

## 1. Introduction

Recently, wind energy as an inexhaustible and fast-growing clean renewable energy source has received considerable attention. As critical equipment for wind power generation, wind turbines have been widely distributed around the world. In practice, these turbines are usually situated in far-flung regions and always suffer from harsh operating environments, which can easily cause various failures and even shutdowns in severe cases [1]. Specifically, sensors, such as pitch angle, power, and speed sensors, which are widely equipped in wind turbines for monitoring and controlling the operation of the entire turbine, are extremely prone to various faults. Statistically, sensor failures account for approximately 15% of the total wind turbine failures [2]. Furthermore, sensor failures can cause signal corruption for condition monitoring and fault diagnosis, which in turn affect the health status of other key subassemblies, thereby reducing the reliability of the turbine and increasing economic losses [3,4]. As a result, it is particularly important and challenging to research effective and valuable fault detection approaches for wind turbine sensors.

Up to now, numerous fault detection techniques for wind turbine sensors have been proposed and discussed. On the one hand, physical-model based approaches have been proved to be effective and commonly adopted, typically including constrained Kalman filter [5], Takagi–Sugeno fuzzy model [6], and observed-based approaches [7,8]. Nevertheless, in practical engineering, it is unrealistic to establish a detailed mathematical model due to the complex electromechanical system construction and highly dynamic operating condition of wind turbines, which limits the further development and application of physical-model based methods, to a large extent. On the other hand, with the advent of advanced sensor technology, instead of requiring physical knowledge or accurate mathematical models, data-driven methods relying only on measured data have become quite attractive in fault detection of wind turbine sensors.

Practically, modern large-scale wind turbines have deployed supervisory control and data acquisition (SCADA) systems to acquire and record a vast amount of rich operational status data [9]. Consequently, due to the availability and economy of SCADA data, sensor fault detection based on SCADA data has been considered as a feasible and valuable method, and, until now, numerous analysis approaches have been extensively studied in the literature. These methods mainly include artificial neural networks (ANN) [10,11], power curve-based method [12,13], support vector machine [14,15,16], and classifier fusion-based method [17]. However, these traditional methods cannot fully capture the complicated non-linear mapping between sensor variables due to their typical shallow structure, and, accordingly, can only achieve limited detection performance [18]. In addition, these studies did not mine the sensor data from the perspective of the multivariate time series, nor was the co-relationship between multivariate was well considered.

Alternatively, as a novel type of machine learning, deep learning has attracted extensive attention from academic and industry community, which aims to extract abstract and valuable information from data via stacking multiple non-linear processing layers in hierarchical architectures, and therefore, is more powerful than those traditional intelligent methods [19]. In recent years, this emerging method has been extensively used in a variety of challenging regression and classification tasks, such as speech recognition [20], affect recognition [21,22], image classification [23], and wind speed prediction [24]. Moreover, deep learning-based wind turbine fault detection and diagnosis has witnessed an emerging research [25,26]. In particular, convolutional deep belief network (CDBN), a powerful hierarchical generative method, was proposed by Lee [27], which has the unique advantages of the weight sharing and nonlinear feature learning and has been successfully introduced into the vibration-based fault diagnosis of bearings [28,29].

However, there are some challenges in applying conventional CDBN to detect wind turbine faults based on SCADA data. Specifically, sensor measurements from wind turbine SCADA systems are multivariate and closely related because of the mutual coupling and interaction between various components in a wind turbine [30]. Moreover, these sensor variables are time series in nature, incorporating the temporal and spatial correlation information simultaneously. This paper proposes a novel classification-based fault detection method for wind turbine sensors. In particular, a multiscale spatio-temporal CDBN (MSTCDBN) is developed to extract the interactions and inherent spatio-temporal characteristics of the SCADA multivariate time series. The proposed method can improve the feature learning capability and, thus, enhance fault detection performance. The main contributions of this paper are summarized as follows.

1. A novel MSTCDBN method is proposed to overcome the limitations of traditional CDBN that lack the ability to capture the spatio-temporal correlations inherent in multivariate time series and cannot realize multiscale feature learning. In other words, the proposed MSTCDBN has the superiorities of spatio-temporal dependence extraction and multiscale feature characterization, simultaneously. At the same time, as far as we know, this is the first time CDBN has been applied to the analysis and processing of multivariate time series.
2. Specifically, the spatio-temporal dependences hidden in the multivariate time series are considered by designing different forms of convolution kernels in a cascade way. Furthermore, the interactive and complementary representations between sensor variables are extracted at multiple different scales of filters in a parallel fashion. The proposed MSTCDBN with the multiscale spatio-temporal feature learning ability enables us to enhance the classification performance greatly.
3. A generic wind turbine benchmark model is utilized to evaluate the effectiveness of the proposed method in the fault detection of wind turbine sensors, and comparative studies are performed.

The reminder of this paper is organized as follows. Section 2 briefly describes the theory of standard CDBN and introduces the proposed MSTCDBN approach for fault detection of wind turbine sensors in detail. A systematic description of the experiment and the acquisition and preprocessing of multivariate time series signals are proposed in Section 3. Section 4 gives the comparative detection results to evaluate the effectiveness of the proposed method. Conclusions are provided in Section 5.

## 2. Proposed MSTCDBN Fault Detection Method

### 2.1. Standard CDBN Methodology

**Figure 1:**
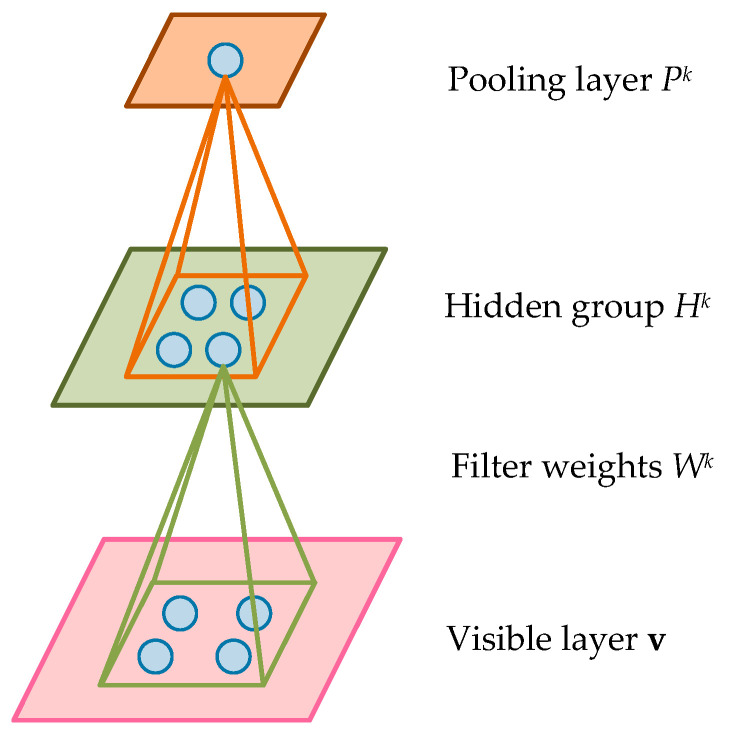
Typical structure of the convolutional restricted Boltzmann machine (CRBM) model.

The standard CDBN is a novel hierarchical probabilistic generative model constituted by several stacked convolutional restricted Boltzmann machines (CRBMs). This model takes the two-dimensional input structure into account and has the superiorities of weight sharing and unsupervised feature learning. Generally, each CRBM contains one visible layer (input layer, typically, binary or Gaussian) and one hidden layer. Since the introduction of the convolution operation, not only the connection weights between these two layers are shared, but also the most significant features of the local area can be extracted. In addition, in order to improve computational efficiency and retain the most useful information, a probabilistic max-pooling layer is usually added after the hidden layer. Figure 1 displays the typical structure of the CRBM model, and for simplicity, only the kth hidden group and the pooling layer are shown.

Assume that the input layer of the CRBM consists of a $N_{V}\times N_{V}$ matrix. The hidden layer is composed of *K* groups with $N_{H}\times N_{H}$ matrix. Therefore, there are $N_{H}^{2}K$ hidden units included in this CRBM. Meanwhile, each hidden group is jointed with a $N_{W}\times N_{W}$ filter, where $N_{W}=N_{V}-N_{H}+1$. In particular, in order to deal with the real-valued input variables of the SCADA system, the Gaussian visible units should be adopted. The energy function of the Gaussian CRBM can be expressed as

(1) $$E(v,h)=\frac{1}{2}\sum_{i,j=1}^{N_{V}}v_{i,j}^{2}-\sum_{k=1}^{K}\sum_{i,j=1}^{N_{H}}\sum_{r,s=1}^{N_{W}}h_{i,j}^{k}W_{r,s}^{k}v_{i+r-1,j+s-1}-\sum_{k=1}^{K}b_{k}\sum_{i,j=1}^{N_{H}}h_{i,j}^{k}-c\sum_{i,j=1}^{N_{V}}v_{i,j},$$

where v and h denote the visible units and hidden units, respectively. $v_{i,j}$ is the element in the ith row and the jth column of the matrix v, $h_{i,j}^{k}$ is the element in the ith row and the jth column of the kth hidden group, and $W_{r,s}^{k}$ is the element in the rth row and sth column of the kth filter. c represents the shared bias of all visible units and $b_{k}$ is the bias of each hidden group.

Then, the conditional distributions of Gaussian CRBMs are calculated according to the block Gibbs sampling [27], which can be described as follows

(2) $$P(h_{i,j}^{k}=1|v)=\sigma ({\left({\tilde{W}}^{k}*v\right)}_{i,j}+b_{k}),$$

(3) $$P(v_{i,j}=1|h)=N(\sum_{k,i,j}{\left(W^{k}*h^{k}\right)}_{i,j}+c,1),$$

where σ(x)=1/(1+exp(x)) represents the logistic sigmoid function, $N(\mu ,\sigma^{2})$ is the Gaussian distribution with mean μ and variance $\sigma^{2}$, ∗ refers to the convolution operation, and ${\tilde{W}}_{i,j}^{k}=W_{N_{W}-j+1}^{k}$.

After obtaining the features of training samples through the convolution operation, the probabilistic max-pooling layer is usually introduced to further reduce the computational complexity and retain the most useful feature information. The pooling layer also has *K* groups, each of which is a $N_{P}\times N_{P}$ matrix. For each k∈{1,⋯,K}, the hidden group $H^{k}$ is partitioned into multiple blocks of size C×C, where C generally refers to a small integer like one, two, or three. Meanwhile, each block is associated with a unit in the pooling layer. Finally, a contrastive divergence method [31] is conducted to get the optimal model parameters $\left\{W^{k},c,b_{k}\right\}$ of the Gaussian CRBM. As a result, the learning process of the hierarchical generative algorithm CDBN can be accomplished by continuously training several individual CRBMs.

### 2.2. MSTCDBN Architecture

**Figure 2:**
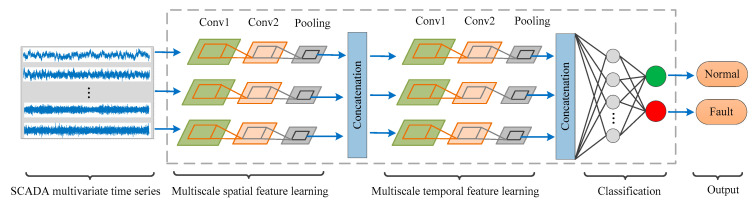
Schematic of the developed multiscale spatio-temporal convolutional deep belief network (MSTCDBN) method.

The main idea of the new MSTCDBN method is to incorporate spatio-temporal characteristic representation and multiscale feature learning into the conventional CDBN structure to enhance fault detection performance. It is worth noting that the fault detection method for wind turbine sensors mentioned in this paper is a classification-based supervised detection method, which typically belongs to a binary classification problem. To be specific, different sensor failure scenarios are uniformly defined as fault condition to perform effective classification detection. The overall schematic is given in Figure 2, and the general implementation process are illustrated as follows.

1. Collect SCADA datasets with various health conditions of wind turbine sensors. For each health condition, data is preprocessed and further divided into several two-dimensional fragments to acquire training and testing sets separately.
2. Multiple CDBN models with different structures are integrated for multiscale spatio-temporal feature learning. In particular, this learning process is realized in a typical unsupervised manner. Then, the obtained multiscale spatio-temporal characteristics are input to a classifier to detect the condition of wind turbine sensors.
3. Testing sets are fed into the well-learned MSTCDBN-based model to perform multiscale spatio-temporal feature extraction and produce detection results.

Inspired by inception structure [32,33], one of the advantages of this approach is to extract and capture useful interactive signatures at multiple different scales of filters in a parallel manner. On the contrary, due to the inherent spatio-temporal characteristics of the multivariate time series, feature extraction along different dimensions can not only make the model more interpretative but also can improve the model performance [34,35]. In view of this, another property is to excavate spatial and temporal correlation information in a cascade way by designing different forms of convolution kernels. In general, the method mainly consists of three consecutive stages, which are multiscale spatial feature learning, multiscale temporal feature leaning, and classification, respectively.

#### 2.2.1. Multiscale Spatial Feature Learning

Given an input sensor measurement matrix $X\in R^{S\times T}$, where s denotes the number of sensor variables, and t represents sampling points. For this multivariate time series matrix, three different scales of filters, including two by one, three by one, and five by one, are used to extract the interactive characteristics among multiple variables in parallel, respectively. Note that each filter is designed to slide only along the variable axis in this section. In other words, three different CDBN modules are initially executed to process the input data, therein extracting advanced abstract multiscale spatial correlation information.

In terms of each CDBN module, it includes two hidden layers followed by a pooling layer, and as mentioned above, the convolution and pooling operations are performed only in the spatial dimension. Once the above process is completed, in order to keep the temporal dimension unchanged, the local spatial feature maps yielded by these CDBN modules are concatenated in the direction of the variable axis for further multiscale temporal feature extraction.

#### 2.2.2. Multiscale Temporal Feature Learning

This phase is designed to extract valuable temporal characteristics from the learned spatial maps at different filter scales in parallel. Similar to the spatial feature learning, three different scales of filters are adopted. However, the difference is that each filter is designed to move only along the time axis of each variable, and the sizes are set to one by two, one by three, and one by five, respectively. This implies that three additional CDBN modules are applied to mine useful and different temporal correlation information. In the same way, each CDBN module contains two hidden layers and a pooling layer. Then, the local temporal correlations extracted at each filter scale are concatenated along the time axis for the final fault classification detection.

#### 2.2.3. Classification

It should be noted that the fault detection for wind turbine sensors focused on this work belongs to the category of binary classification, which indicates whether the sensors are in a healthy condition. In this case, the high-level multiscale spatio-temporal representations learned in the feature extraction phase is first transformed into a two-dimensional matrix, and then directly fed into the final softmax function to convert each class of predictions into conditional probabilities. For the training of the proposed method, the cross entropy function is selected as the loss function, as shown in Equation (4), where p(i) refers to the true distribution, and q(i) stands for the estimated distribution. Finally, after adequate training of the proposed method, the testing set is further used for performance evaluation.

(4) $$H(p,q)=-\sum_{i}p(i)\log q(i),$$

## 3. Case Study

### 3.1. Available Data

**Figure 3:**
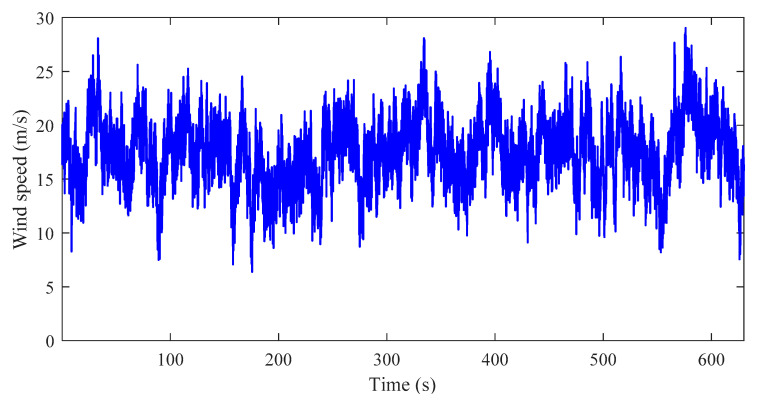
Wind speed sequence with mean speed of 17 m/s.

In order to implement the proposed approach in wind turbine sensor fault detection, a generic 5MW-based offshore wind turbine benchmark model presented in [36] was employed. This benchmark models a realistic three-bladed, variable speed horizontal axis wind turbine using a fatigue, aerodynamics, structures, and turbulence (FAST) aeroelastic simulator, and has been extensively applied to evaluate a variety of fault detection and diagnostic approaches [37,38]. Moreover, the model can generate actual stochastic wind data series, and the cut-in, rated, and cut-out wind speeds were 3 m/s, 11.4 m/s, and 25 m/s, respectively. A more detailed illustration of the wind turbine benchmark model was given in [36]. In this work, in order to verify the proposed approach in a more realistic scenario, a mean speed of 17 m/s at hub height was used to generate the required data set, and a simulation of this wind speed sequence is shown in Figure 3.

In a wind turbine SCADA system, vast amounts of measurements were collected to monitor the operating status of the turbine and its key subsystems, such as power output, wind speed, temperature, blade pitch angle, and generator speed. Similarly, in regard to the benchmark model, a total of 15 sensor outputs with measurement noise are provided, as listed in Table 1 [17], each of which was generated by adding a band-limited Gaussian white noise that are parameterized by noise power to the actual variable value by the FAST simulator. Note that all these measured variables were derived from real wind turbine SCADA systems. Based on this benchmark model, the actual sensor failure scenarios of wind turbines can also be defined. Overall, six sensor operating conditions with different kinds of faults were involved in this work, and the details of all these conditions are displayed in Table 2.

### 3.2. Data Collection and Preprocessing

In this section, in order to acquire more realistic time series measurements, different wind data sets were used for generating numerical simulations. Note that the sampling time of each data set was 0.0125 s, and the duration was 630 s. In particular, 420 simulations were carried out totally, of which 210 were in normal condition and 45 for each failure scenario.

However, in practice, the sampling time of 0.0125 s was usually relatively low compared with the real SCADA system. Therefore, in order to consider a realistic higher sampling time [39], the raw measured values from all conditions were down-sampled to 1 s. Furthermore, because the initialization of the numerical simulation can result in transients [40], the first 30 s sequence of each simulation was deleted. Generally, different variables had different dimensions, so it was necessary to normalize these measurements such that they are in a consistent range of [0,1]. After that, in order to train the proposed method on the multivariate time series, the normalized signals were divided into a set of segments with a length of 100 sampling points without overlap through using sliding window technology, which meant that each time series segment represents a sequence of 100 s. At this point, there were 1260 samples for the normal condition and 270 samples for each failure scenario, and these samples were manually labeled to indicate whether the sensor was in a healthy condition. In this paper, a commonly used random selection approach was used for performance evaluation, which can avoid the impact of contingency and particularity on the diagnosis results. In addition, considering that data bias could severely deteriorate the evaluation results, these data sets were intentionally divided to make the classes balanced. In particular, random 1000 samples of the normal condition were selected for model training and 250 samples for testing. For each fault condition, random 200 samples were applied for training and 50 samples for testing. The detailed description of the sample distribution is shown in Table 3, and the dimension of each multivariate time series sample was 15 by 100.

## 4. Results

In this section, in order to verify the effectiveness of the proposed approach and overcome the influence of randomness in the model training process on the detection results, ten trials were conducted for evaluating the overall performance. Meanwhile, the advantage of the proposed MSTCDBN fault detection method was proved by comparing with traditional CDBN and its other variants. Here, four commonly adopted evaluation metrics, classification accuracy, precision, recall, and F1 score were used for performance evaluation and comparison, which can be defined as follows

(5) $$\mathrm{Accuracy}=\frac{\mathrm{TP}+\mathrm{TN}}{\mathrm{TP}+\mathrm{FN}+\mathrm{FP}+\mathrm{TN}},$$

(6) $$\mathrm{Precision}=\frac{\mathrm{TP}}{\mathrm{TP}+\mathrm{FP}},$$

(7) $$\mathrm{Recall}=\frac{\mathrm{TP}}{\mathrm{TP}+\mathrm{FN}},$$

(8) $$F1-\mathrm{score}=\frac{2\mathrm{Precision}\times \mathrm{Recall}}{\mathrm{Precision}+\mathrm{Recall}},$$

where TP refers to the number of correctly classified as positive samples, TN is the number of correctly classified as negative samples, FP is the number of misclassified as positive samples, and FN is the number of misclassified as negative samples, respectively.

As described in Section 2, in order to extract interactive spatio-temporal features for sensor fault detection, six different forms of CDBN were designed in the proposed MSTCDBN method. Each CDBN module consists of two hidden layers and a pooling layer, and the number of filter groups for two hidden layers were set to 9 and 16, respectively. The detailed structures are listed in Table 4. In addition, in the process of model training, the batch sizes of each CDBN were 100 and 10, and the stride length was selected as 1 by 1. In the final classification phase, the output size of the MSTCDBN was two, which corresponds to the normal and fault conditions of the sensor, respectively.

**Figure 4:**
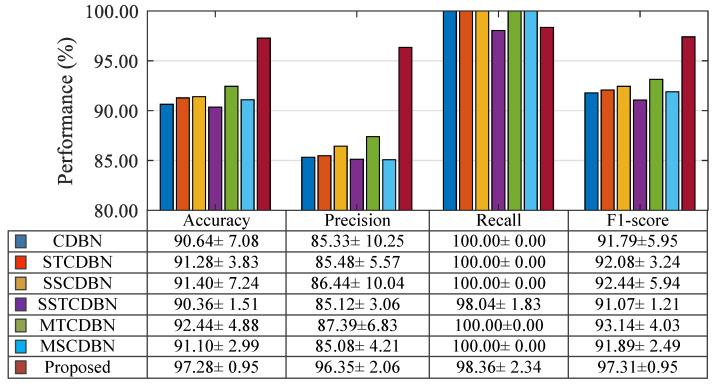
Detection performance of different convolutional deep belief network (CDBN) structures.

In addition, herein, several structures of CDBN from different perspectives were investigated to deeply explore the capability of the presented method in the wind turbine sensor fault detection, including standard CDBN, single-scale temporal CDBN (STCDBN), single-scale spatial CDBN (SSCDBN), single-scale spatio-temporal CDBN (SSTCDBN), multiscale temporal CDBN (MTCDBN), and multiscale spatial CDBN (MSCDBN). Specifically, the first three methods all contain one CDBN module and only consider the correlations on a single scale. For the standard CDBN, conventional square filters are adopted. The filters of STCDBN and SSCDBN are specified to slide only along the time and spatial axes, respectively. In terms of the SSTCDBN, it contains two cascaded CDBN modules, taking into account the correlations in both temporal and spatial dimensions on a single scale. For the latter two methods, MTCDBN and MSCDBN consist of three parallel CDBN modules, extracting temporal and spatial information on multiple scales, respectively. In these experiments, the same input, two-layer structures CDBN and softmax classifier as the proposed method was used, and the stride length and batch sizes were also set to 1 by 1 and 100 and 10, respectively. The detailed structures are shown in Table 5. Moreover, all models with the running environment Intel Core (TM) i5-4300 CPU and 8-GB RAM using the Matlab software. The average testing performance (mean ± standard deviation) of all methods over ten trials are given in Figure 4 and the average testing time is shown in Table 6.

It can be easily seen from Figure 4 that the first six methods yield similar detection performance. However, compared with standard CDBN with conventional square filter, STCDBN and SSCDBN have better performance, which shows the ability of temporal and spatial information extraction. Moreover, in terms of the mean value, it can be seen that MTCDBN is superior to STCDBN in all evaluation metrics. Although MSCDBN is slightly inferior to SSCDBN, the proposed method obviously outperforms these two methods and other structures of CDBN. This is mainly because MSTCDBN with different scales of filters can extract and learn the interactive spatio-temporal correlation information that is beneficial for classification. Specifically, better and more stable improvements from other variants of CDBN to MSTCDBN can be observed for accuracy, precision, and F1-score. Overall, the method presented in this paper results in an enhanced fault detection performance. Likewise, it is not difficult to find from Table 6 that although the structure of the proposed method is relatively complex, it costs less computing time than the MTCDBN and MSCDBN due to the introduction of pooling operation and need for the fewer number of filter groups.

**Figure 5:**
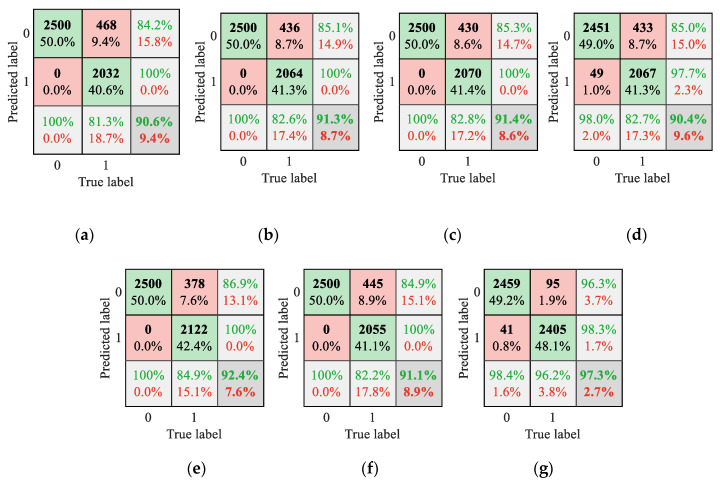
Confusion matrix results of different methods. (**a**) standard CDBN; (**b**) single-scale temporal CDBN (STCDBN); (**c**) single-scale spatial CDBN (SSCDBN); (**d**) single-scale spatio-temporal CDBN (SSTCDBN); (**e**) multiscale temporal CDBN (MTCDBN); (**f**) multiscale spatial CDBN (MSCDBN); and (**g**) proposed method.

From another perspective, in order to better understand the classification performance of different approaches, the testing classification results over ten trials using the confusion matrix are given in Figure 5. The 0 and 1 represent normal and fault conditions, respectively. It can be observed that the confusion matrix comprehensively describes the number of correctly classified samples and misclassified samples for normal and fault conditions, the percentage of each condition that is correctly classified and incorrectly classified, and the percentage of correctly classified and misclassified in each predicted label. Obviously, compared with other methods, fewer total samples are misclassified when employing proposed MSTCDBN method, resulting in better detection performance.

According to Section 3, six health conditions of sensors are mainly focused on this paper, including the normal condition and five different patterns of faults. Therefore, it is necessary to quantitatively evaluate the detection performance of an individual condition based on the overall detection results. The comparative results with average classification accuracy are given in Table 7.

As can be observed from Table 7 that in terms of normal condition, fault types three and four, all methods achieved relatively good performance, with the classification accuracy of above 98%, 93%, and 92%, respectively. Moreover, the proposed method and SSTCDBN outperform the other models in classifying fault type one. As far as fault type five was concerned, the recognition accuracy of MSCDBN was only 62.8%, which is significantly lower than other methods. It is worth mentioning that as for fault type two, the approach presented in this paper achieved the highest classification accuracy of 83.6%. However, with respect to other variants of CDBN, the best result was only 60.4%, which clearly shows the advantage of the proposed method in fault identification. Moreover, it also indirectly indicates that fault type two is probably the most difficult of the six health conditions to detect. In a word, the method incorporating multiscale and spatio-temporal feature learning capability presented in this paper plays an important role in sensor fault detection and finally obtains the highest overall accuracy. 

In order to further demonstrate the ability of the proposed method in fault detection of wind turbine sensors, a comparison with traditional ANN and deep belief network (DBN) is carried out. For these two networks, the four-layer structures consisting of an input layer, two hidden layers, and an output layer are used for classification. It should be noted that different from the CDBN model, DBN, and ANN deal with data on the one-dimensional input structure. Table 8 shows the average comparison results between the proposed MSTCDBN and the two methods in terms of four evaluation metrics over ten trails. 

It can be easily found from Table 8 that the accuracy, precision, recall, and F1-score generated by the proposed MSTCDBN method was higher than the ANN and DBN models. It means that the proposed method achieves the best overall performance compared to the other two methods, indicating the superiority of the proposed method in fault detection. Furthermore, it can also be seen that the DBN performs better than the ANN. This is mainly because compared with the ANN model, DBN has powerful unsupervised feature learning ability, which can handle complex relationships between variables, thus resulting in relatively better performance. However, this model ignores the two-dimensional structure of the input, making it difficult to extract the spatio-temporal correlations hidden in the multivariate time series. In contrast, the proposed MSTCDBN method takes into account spatio-temporal features at different filter scales, which contributions to the enhancement of the fault detection performance. 

In summary, since the sensor measurements generated by the benchmark model can truly reflect the wind turbine SCADA data, the proposed MSTCDBN method has the potential to be an alternative method for sensor fault detection in real wind farms. Meanwhile, in practice, it is reasonable to train an independent model for each turbine due to the different operating conditions and environments of each turbine. Likewise, the condition labels which are helpful for fault detection should be carefully labeled so that the proposed method is widespread adopted.

## 5. Conclusions

In this paper, a novel MSTCDBN approach is presented to address the challenging task of fault detection of wind turbine sensors by considering the temporal and spatial correlations hidden in the multivariate time series. Firstly, a major feature of the presented approach is that different forms of convolution kernels are designed to extract the spatio-temporal characteristics between sensor variables in a cascade way. Secondly, another contribution is to learn interactive and rich fault features by incorporating different scales of filters in a parallel manner. Accordingly, the proposed method can enhance the feature learning capacity and fault detection performance. Furthermore, the effectiveness of the MSTCDBN model was investigated by comparing with traditional CDBN and its other variants, as well as ANN and DBN methods. In particular, the developed method combing spatio-temporal feature extraction and multiscale learning is better than other models in terms of classification performance, which provides a new insight for detecting wind turbine sensor faults and can be extended to other research applications.

However, due to the limited SCADA data, this study is only validated on a generic benchmark model. In future work, the developed MSTCDBN will further be spread to real wind turbine sensors, and other critical components in wind turbines will be discussed. From the perspective of pattern recognition, a fault diagnosis system can quickly identify and locate fault categories when abnormalities occur in the wind turbine. Therefore, the proposed method will be further used for the sensor fault diagnosis to identify the specific fault type of the sensor. Based on the sensor fault detection and diagnosis, it is necessary to adopt corresponding fault-tolerant control strategies to ensure that the turbine is in normal operation, thereby improving the safety and reliability of the turbine. Meanwhile, more advanced feature extraction and learning approaches will be established to further mine the spatio-temporal correlations in SCADA multivariate time series. In this paper, the data-driven method is used to realize the fault detection, and the combination of data-driven and model-based methods is expected to become a new research direction [41]. 

## Figures and Tables

**Table 1:** Available variables.

Number	Variable	Unit	Noise Power
1	Wind speed at hub height	m/s	0.0071
2	Rotor speed	rad/s	$10^{-4}$
3	Generator speed	rad/s	2 × $10^{-4}$
4	Generator torque	Nm	0.9
5	Generated electrical power	W	10
6, 7, 8	Pitch angle of ith blade	deg	1.5 × $10^{-3}$
9	Azimuth angle low speed	rad	$10^{-3}$
10, 11, 12	Blade root moment of ith blade	Nm	$10^{3}$
13, 14	Tower acceleration in *x*, *y* direction	${m/s}^{2}$	5 × $10^{-4}$
15	Yaw error	deg	5 × $10^{-2}$

**Table 2:** Detailed descriptions of considered conditions.

Number	Condition	Description
Normal	Normal condition	Fault-free
Fault 1	Generator speed sensor	Scaling (gain factor equal to 1.2)
Fault 2	Generator power sensor	Scaling (gain factor equal to 1.2)
Fault 3	Pitch angle sensor	Stuck (fixed value equal to 1 deg)
Fault 4	Pitch angle sensor	Stuck (fixed value equal to 5 deg)
Fault 5	Pitch angle sensor	Scaling (gain factor equal to 1.2)

**Table 3:** Sample description of the different sensor conditions.

Number	Size of Total Samples	Size of Training Samples	Size of Testing Samples
Normal	1260	1000	250
Fault 1	270	200	50
Fault 2	270	200	50
Fault 3	270	200	50
Fault 4	270	200	50
Fault 5	270	200	50

**Table 4:** The structure of the proposed MSTCDBN.

Description	Setting	Convolution 1	Convolution 2	Pooling
Multiscale spatial feature learning	CDBN1	9, 2×1	16, 2×1	2×1
	CDBN2	9, 3×1	16, 3×1	2×1
	CDBN3	9, 5×1	16, 5×1	2×1
Multiscale temporal feature learning	CDBN4	9, 1×2	16, 1×2	1×2
	CDBN5	9, 1×3	16, 1×3	1×2
	CDBN6	9, 1×5	16, 1×5	1×2

**Table 5:** Structures of six comparative methods.

Method	Setting	Convolution 1	Convolution 2	Pooling
CDBN	CDBN1	16, 3×3	32, 3×3	2×2
STCDBN	CDBN1	9, 1×5	16, 1×5	1×2
SSCDBN	CDBN1	16, 2×1	16, 2×1	-
SSTCDBN	CDBN1	9, 2×1	16, 2×1	2×1
	CDBN2	9, 1×2	16, 1×2	1×2
MTCDBN	CDBN1	16, 1×2	16, 1×2	1×2
	CDBN2	16, 1×3	16, 1×3	1×2
	CDBN3	16, 1×5	16, 1×5	1×2
MSCDBN	CDBN1	32, 2×1	32, 2×1	-
	CDBN2	32, 3×1	32, 3×1	-
	CDBN3	32, 5×1	32, 5×1	-

**Table 6:** Runtime of different CDBN structures.

Method	Time (s)	Method	Time (s)
CDBN	23.33	MTCDBN	83.60
STCDBN	13.77	MSCDBN	105.20
SSCDBN	20.36	Proposed method	56.27
SSTCDBN	10.62		

**Table 7:** Recognition accuracy of each health condition (%).

Method	Normal	Fault 1	Fault 2	Fault 3	Fault 4	Fault 5	Overall
CDBN	100.00	76.20	44.60	93.40	92.40	99.80	90.64
STCDBN	100.00	90.00	33.00	95.40	94.40	100.00	91.28
SSCDBN	100.00	81.00	41.20	97.00	94.80	100.00	91.40
SSTCDBN	98.04	99.80	19.40	99.00	99.60	95.60	90.36
MTCDBN	100.00	83.60	50.60	94.80	100.00	95.40	92.44
MSCDBN	100.00	96.60	60.40	95.80	95.40	62.80	91.10
Proposed	98.36	99.20	83.60	98.60	99.60	100.00	97.28

**Table 8:** Comparison results of other different methods (%).

Method	Accuracy	Precision	Recall	F1-Score
ANN	74.13	78.19	69.49	87.02
DBN	88.64	86.05	95.78	90.03
Proposed	97.28	96.35	98.36	97.31

## References

[1] Shafiee M. (2015). Maintenance logistics organization for offshore wind energy: Current progress and future perspectives. Renew. Energy.

[2] Qiao W., Lu D. (2015). A survey on wind turbine condition monitoring and fault diagnosis-Part I: Components and subsystems. IEEE Trans. Ind. Electron..

[3] Peng Y., Qiao W., Qu L., Wang J. (2018). Sensor fault detection and isolation for a wireless sensor network-based remote wind turbine condition monitoring system. IEEE Trans. Ind. Appl..

[4] Alizadeh E., Meskin N., Khorasani K. (2018). A dendritic cell immune system inspired scheme for sensor fault detection and isolation of wind turbines. IEEE Trans. Ind. Inform..

[5] Nabil E., Sobaih A.A., Abou-Zalam B. Constrained Kalman filter based detection and isolation of sensor faults in a wind turbine. Proceedings of the 10th International Conference on Computer Engineering & System.

[6] Abdelmalek S., Barazane L., Larabi A., Bettayeb M. (2016). A novel scheme for current sensor faults diagnosis in the stator of a DFIG described by a T-S fuzzy model. Measurement.

[7] Wei X., Verhaegen M. (2011). Sensor and actuator fault diagnosis for wind turbine systems by using robust observer and filter. Wind Energy.

[8] Sanchez H., Escobet T., Puig V., Odgaard P. (2015). Fault diagnosis of an advanced wind turbine benchmark using interval-based ARRs and observers. IEEE Trans. Ind. Electron..

[9] Tautz-Weinert J., Watson S.J. (2017). Using SCADA data for wind turbine condition monitoring—A review. IET Renew. Power Gen..

[10] Kavaz A.G., Barutcu B. (2018). Fault Detection of wind turbine sensors using artificial neural networks. J. Sens..

[11] Zhang Z., Wang K. (2014). Wind turbine fault detection based on SCADA data analysis using ANN. Adv. Manuf..

[12] Pei Y., Qian Z., Jing B., Kang D.H., Zhang L.Z. (2018). Data-driven method for wind turbine yaw angle sensor zero-point shifting fault detection. Energies.

[13] Schlechtingen M., Santos I.F., Achiche S. (2013). Using data-mining approaches for wind turbine power curve monitoring: A comparative study. IEEE Trans. Sustain. Energy.

[14] Laouti N., Othman S., Alamir M., Sheibat-Othman N. (2014). Combination of model-based observer and support vector machines for fault detection of wind turbines. Int. J. Autom. Comput..

[15] Vidal Y., Pozo F., Tutiven C. (2018). Wind turbine multi-fault detection and classification based on SCADA Data. Energies.

[16] Leahy K., Hu R.L., Konstantakopoulos L.C., Spanos C.J., Agogino A.M. Diagnosing wind turbine faults using machine learning techniques applied to operational data. Proceedings of the 2016 IEEE International Conference on Prognostics and Health Management.

[17] Pashazaden V., Salmasi F.R., Araabi B.N. (2018). Data driven sensor and actuator fault detection and isolation in wind turbine using classifier fusion. Renew. Energy.

[18] Bengio Y., Courville A., Vincemt P. (2013). Representation learning: A review and new perspectives. IEEE Trans. Pattern Anal. Mach. Intell..

[19] Zhao R., Yan R., Chen Z., Mao K., Wang P., Gao R.X. (2019). Deep learning and its applications to machine health monitoring. Mech. Syst. Signal Proc..

[20] Mustaqeem, Kwon S. (2020). A CNN-assisted enhanced audio signal processing for speech emotion recognition. Sensors.

[21] Sun B., Cao S., He J., Yu L. (2018). Affect recognition from facial movements and body gestures by hierarchical deep spatio-temporal features and fusion strategy. Neural Netw..

[22] Zhang Q., Chen X., Zhan Q., Yang T., Xia S. (2017). Respiration-based emotion recognition with deep learning. Comput. Ind..

[23] Wang Y., Wang X., Liu W. (2016). Unsupervised local deep feature for image recognition. Inf. Sci..

[24] Wan J., Liu J., Ren G., Guo Y., Yu D., Hu Q. (2016). Day-ahead prediction of wind speed with deep feature learning. Int. J. Pattern Recognit. Artif. Intell..

[25] Jiang G., He H., Yan J., Xie P. (2019). Multiscale convolutional neural networks for fault diagnosis of wind turbine gearbox. IEEE Trans. Ind. Electron..

[26] Jiang G., Xie P., He H., Yan J. (2018). Wind turbine fault detection using a denoising autoencoder with temporal information. IEEE ASME Trans. Mechatron..

[27] Lee H., Grosse R., Ranganath R., Ng A.Y. (2011). Unsupervised learning of hierarchical representations with convolutional deep belief networks. Commun. ACM.

[28] Shao H., Jiang H., Zhang H., Liang T. (2018). Electric locomotive bearing fault diagnosis using a novel convolutional deep belief network. IEEE Trans. Ind. Electron..

[29] Shao H., Jiang H., Zhang H., Duan W., Liang T., Wu S. (2018). Rolling bearing fault feature learning using improved convolutional deep belief network with compressed sensing. Mech. Syst. Signal Proc..

[30] Yang W., Court R., Jiang J. (2013). Wind turbine condition monitoring by the approach of SCADA data analysis. Renew. Energy.

[31] Hinton G.E. (2002). Training products of experts by minimizing contrastive divergence. Neural Comput..

[32] Szegedy C., Liu W., Jia Y., Sermanet P., Reed S., Anguelov D. Going deeper with convolutions. Proceedings of the IEEE Conference on Computer Vision and Pattern Recognition.

[33] Liu C., Hsaio W.H., Tu Y. (2019). Time series classification with multivariate convolutional neural network. IEEE Trans. Ind. Electron..

[34] Gao Z., Wang X., Yang Y., Mu C., Cai Q., Dang W. (2019). EEG-based spatio-temporal convolutional neural network for driver fatigue evaluation. IEEE Trans. Neural Netw. Learn. Syst..

[35] Zhang D., Yao L., Chen K., Wang S., Chang X., Liu Y. (2019). Making sense of spatio-temporal preserving representations for EEG-based human intention recognition. IEEE Trans. Cybern..

[36] Odgaard P.F., Johnson K.E. Wind turbine fault detection and fault tolerant control-An enhanced benchmark challenge. Proceedings of the America Control Conference.

[37] Yang W., Liu C., Jiang D. (2018). An unsupervised spatiotemporal graphical modeling approach for wind turbine condition monitoring. Renew. Energy.

[38] Ruiz M., Mujica L.E., Alferez S., Acho L., Tutiven C., Vidal Y., Rodellar J., Pozo F. (2018). Wind turbine fault detection and classification by means of image texture analysis. Mech. Syst. Signal Proc..

[39] Gonzalez E., Stephen B., Infield D., Melero J.J. (2017). On the use of high-frequency SCADA data for improved wind turbine performance monitoring. J. Phys. Conf. Ser..

[40] Lackner M.A., Rotea M.A. (2011). Passive structural control of offshore wind turbines. Wind Energy.

[41] Chan J.C.L., Tan C.P., Trinh H., Kamal M.A.S. (2019). State and fault estimation for a class of non-infinitely observable descriptor systems using two sliding mode observers in cascade. J. Frankl. Inst.-Eng. Appl. Math..

